# Clinical Validation of a Machine-Learned, Point-of-Care System to IDENTIFY Functionally Significant Coronary Artery Disease

**DOI:** 10.3390/diagnostics14100987

**Published:** 2024-05-08

**Authors:** Thomas D. Stuckey, Frederick J. Meine, Thomas R. McMinn, Jeremiah P. Depta, Brett A. Bennett, Thomas F. McGarry, William S. Carroll, David D. Suh, John A. Steuter, Michael C. Roberts, Horace R. Gillins, Farhad Fathieh, Timothy Burton, Navid Nemati, Ian P. Shadforth, Shyam Ramchandani, Charles R. Bridges, Mark G. Rabbat

**Affiliations:** 1 Cone Health Heart and Vascular Center, Greensboro, NC 27401, USA; thomas.stuckey@conehealth.com; 2 Novant Health New Hanover Regional Medical Center, Wilmington, NC 28401, USA; frederick.meine@novanthealth.org; 3 Austin Heart, Austin, TX 78705, USA; 4 Rochester General Hospital, Rochester, NY 14621, USA; 5 Jackson Heart Clinic, Jackson, MS 39216, USA; 6 Oklahoma Heart Hospital, Oklahoma City, OK 73120, USA; 7 Cardiology Associates of North Mississippi, Tupelo, MS 38801, USA; 8 Atlanta Heart Specialists, Tucker, GA 30084, USA; 9 Bryan Heart, Lincoln, NE 68506, USA; 10 Lexington Medical Center Heart & Vascular, West Columbia, SC 29169, USA; 11 CorVista Health, Inc., Bethesda, MD 20814, USAcbridges@corvista.com (C.R.B.); 12 Analytics for Life, Inc., Toronto, ON M5X 1C9, Canadatim@analytics4life.com (T.B.); navid.nemati@analytics4life.com (N.N.); shyam@analytics4life.com (S.R.); 13 Loyola University Medical Center, Maywood, IL 60153, USA

**Keywords:** machine learning, artificial intelligence, coronary artery disease, digital health, front-line testing

## Abstract

Many clinical studies have shown wide performance variation in tests to identify coronary artery disease (CAD). Coronary computed tomography angiography (CCTA) has been identified as an effective rule-out test but is not widely available in the USA, particularly so in rural areas. Patients in rural areas are underserved in the healthcare system as compared to urban areas, rendering it a priority population to target with highly accessible diagnostics. We previously developed a machine-learned algorithm to identify the presence of CAD (defined by functional significance) in patients with symptoms without the use of radiation or stress. The algorithm requires 215 s temporally synchronized photoplethysmographic and orthogonal voltage gradient signals acquired at rest. The purpose of the present work is to validate the performance of the algorithm in a frozen state (i.e., no retraining) in a large, blinded dataset from the IDENTIFY trial. IDENTIFY is a multicenter, selectively blinded, non-randomized, prospective, repository study to acquire signals with paired metadata from subjects with symptoms indicative of CAD within seven days prior to either left heart catheterization or CCTA. The algorithm’s sensitivity and specificity were validated using a set of unseen patient signals (*n* = 1816). Pre-specified endpoints were chosen to demonstrate a rule-out performance comparable to CCTA. The ROC-AUC in the validation set was 0.80 (95% CI: 0.78–0.82). This performance was maintained in both male and female subgroups. At the pre-specified cut point, the sensitivity was 0.85 (95% CI: 0.82–0.88), and the specificity was 0.58 (95% CI: 0.54–0.62), passing the pre-specified endpoints. Assuming a 4% disease prevalence, the NPV was 0.99. Algorithm performance is comparable to tertiary center testing using CCTA. Selection of a suitable cut-point results in the same sensitivity and specificity performance in females as in males. Therefore, a medical device embedding this algorithm may address an unmet need for a non-invasive, front-line point-of-care test for CAD (without any radiation or stress), thus offering significant benefits to the patient, physician, and healthcare system.

## 1. Introduction

85 million Americans reside in rural areas, which are significantly underserved by the healthcare system, leading to disparities in health outcomes compared to urban populations. The healthcare provider gap is evident, with 44% more primary care physicians available per 100,000 people located in urban areas compared to rural areas [1]. The situation is not improving, as demonstrated by the closure of more than 100 rural hospitals, representing 4% of the total, between 2013 and 2020 [2]. When rural patients receive care, their disease presentation is often much more advanced than that typically seen in urban settings, indicating delayed access to care. This delay is often exacerbated due to the long distances required to travel to appointments [3]. Specifically, only 31% of rural residents are within 20 km of an interventional cardiologist (as compared to 87% of urban residents), 19% of an electrophysiologist (78% for urban) and 5% of a heart failure specialist (48% for urban) [4]. The reduced access translates clearly into outcomes, with the age-adjusted (rural population is older) all-cause mortality being 18% higher than the urban population [1]. However, rural patients of all ages are impacted, with younger rural patients experiencing notably higher mortality from coronary artery disease (CAD) than their urban counterparts [5]. 

Current diagnostic techniques for CAD are costly and inconvenient, and they expose the patient to risks. Several studies have evaluated the performance of the diagnostic tests routinely utilized to assess CAD, such as a typical front-line test exercise ECG. However, the studies are challenging to compare, given, for instance, the variation in subject entrance criteria and differing disease definitions. The result is a large range in the reported performance, including those cited in the ACC guidelines [6]. In one case, a meta-analysis including 147 studies and comprising 24,074 individuals who had both an exercise ECG and an invasive coronary angiography (ICA) found that exercise ECG had an average sensitivity of 68% (range of 23–100%) and an average specificity of 77% (range of 17–100%) [7]. 

While the definition of “significant CAD” varies, ACC guidelines classify a significant lesion as any lesion ≥50% in the left main coronary artery (LMCA) and ≥70% in the left circumflex (LCX), left anterior descending (LAD), ramus, and right coronary (RCA) arteries or their distributions. Further, functional measures of lesion significance are increasingly being used in practice, with one such measure being fractional flow reserve (FFR), whereby an FFR ≤ 0.80 is significant [8]. Instantaneous wave-free ratio (iFR) is a similar functional measurement, with a significance threshold at iFR ≤ 0.89. Indeed, as the specific lesion morphology and collaterals have a significant impact, it is unsurprising that a significant occlusion as defined by an anatomic arterial narrowing of >50% is prognostic of a functionally significant occlusion (FFR ≤ 0.80) only 68% of the time [9].

Given the wide ranges of sensitivity and specificity to identify CAD, test safety is critically relevant. Regardless of whether the first assessment of patients with symptoms that may be indicative of obstructive CAD (new onset chest pain, etc.) is with functional testing, such as single photon emission computed tomography (SPECT) or coronary computed tomography angiography (CCTA), 90% will ultimately have negative findings [8]. Here, a negative test typically augurs a superb long-term outcome (NPV > 96%) [10]. In addition, patients who initially test positive usually receive additional assessment. In patients who undergo invasive coronary angiography (ICA), the gold standard to confirm the presence of CAD, only 32–42% truly have significant CAD [10,11]. Therefore, taken in series, significant CAD is ultimately diagnosed in only 3–4% of patients who initially presented with symptoms of obstructive CAD [10].

Clinically, although the initial CAD likelihood is low in symptomatic patients, it is critical to detect the higher-risk subgroup. Consequently, sensitivity is usually sought over specificity. Clinicians have frequently employed tests with an average sensitivity and specificity of approximately 70% with a lower confidence interval of 60% or less [10,12]. This clinical utilization relies on the NPV being relatively high given the low disease prevalence, and further, patients with test-positive results will typically undergo additional testing, which best mitigates patient risk. 

The most used test for suspected CAD in the USA is SPECT, representing almost 80% of initial testing for CAD. A meta-analysis of non-invasive tests for CAD reported the sensitivity of SPECT to be 73% (62–82%) and specificity to be 83% (71–90%) [9]; with high specificity that exceeds sensitivity, SPECT is, therefore, a rule-in test. Conversely, the reported performance of CCTA in identifying functionally significant CAD is a sensitivity of 93% (89–96%) and specificity of 53% (37–68%) [9]; with high sensitivity that exceeds specificity, CCTA is, therefore, a rule-out test. CCTA consequently has an exceptionally high NPV, over 98%, assuming a 4% disease prevalence. Knuuti et al. suggest that “With low prevalence of CAD the primary first task of imaging may be the accurate exclusion of anatomic CAD, for which CCTA has demonstrated a strong role.” [9]. 

However, in the USA, CCTA makes up only 2% of initial tests, limited by its lack of availability within a reasonable distance of where many patients live [13]. Though patients from both urban and rural locales have difficulty in accessing CCTA, rural patients suffer from particularly poor access. For instance, CCTA is available in only 22% of rural safety-net hospitals, as compared to 57% in urban settings [12]. Further, only 7.7% of small centers (6 to 49 beds), more typical of rural vs. urban care, provide CCTA, as compared to 88.9% of large centers (at least 400 beds) [12]. Since travel to CCTA testing can be substantial in rural settings, patients referred for this testing often do not present for their appointment, and therefore, the diagnostic yield is reduced proportionally in this demographic. Consequently, there would be significant benefits to patients, physicians, and the healthcare system if there were a test that had similar performance to CCTA but which was readily available at the point of care with results immediately available, minimizing the probability that patients would be lost to follow-up. 

We have previously designed a machine-learned algorithm to assess for the presence of significant CAD using a non-invasive signal acquired with a portable device requiring only an internet connection [14]. The test combines hardware with low capital cost and cloud-based processing, along with point-of-care viewing of reports to enable access to both the test and healthcare practitioners for rapid interpretation to any site that has WiFi or cellular coverage. Therefore, the test is capable of serving the vast majority of the USA and is particularly well suited to addressing the needs of the rural population. 

The purpose of the present study is the validation of the algorithm (in a static, frozen state, i.e., without any retraining) on a large blinded dataset. The primary statistical validation endpoints have been set to ensure a front-line test comparable to CCTA, with the lower confidence bounds for sensitivity and specificity set to 0.80 and 0.40, respectively. 

The authors completed the STARD checklist [15] for reporting diagnostic performance studies to ensure robust coverage (Supplement Section S1). 

## 2. Materials and Methods

### 2.1. Clinical Data

IDENTIFY (NCT03864081, approved by the Western Institutional Review Board, published on clinicaltrials.gov) is a multicenter, prospective, non-randomized, ongoing repository study designed to acquire physiological signals along with subject metadata from subjects with cardiovascular symptoms indicative of obstructive CAD (see Supplement Section S2 for inclusion/exclusion criteria). IDENTIFY enrolled subjects for the development of machine-learned algorithms, followed by subsequent validation. All subjects provided informed consent.

The validation population consisted of subjects consecutively enrolled from 31 July 2019 through 29 September 2022 (*n* = 1816) across 20 clinical sites (Supplement Section S3). None of the subjects used in validation were available to the algorithm development team nor used in algorithm development. Blinding based on enrollment date implements a higher standard of validation than random selection as it better mimics real-world use of the algorithm, and it is possible for clinical sites to participate only in development or only in validation (Supplement Section S3). Thus, any peculiarities of a particular clinical site may only be captured in either development or validation, increasing the generalizability of the test. 

### 2.2. Signal Capture Device

**Figure 1:**
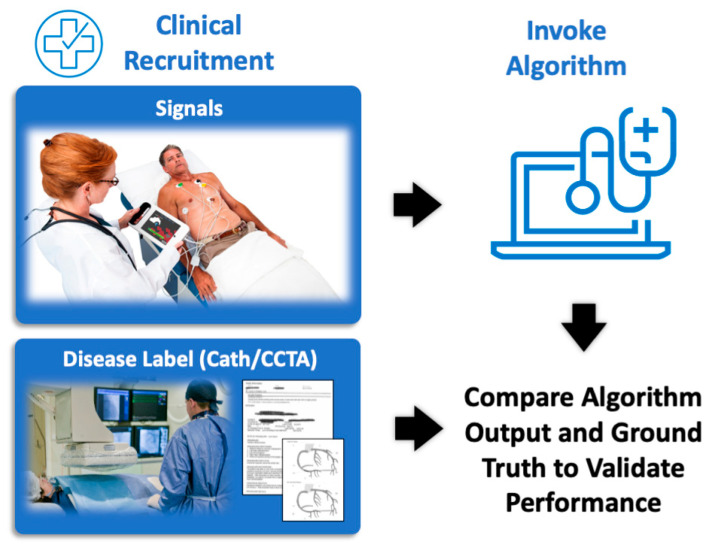
Process for validating machine-learned algorithm. The CAD algorithm is validated by first using a proprietary capture device to collect a suitably large set of patient signals representing the intended use population. The ground truth CAD label (positive or negative) is also collected for each patient. The signals are processed by the machine-learned algorithm. The output scores from the algorithm are converted into binary outputs, CAD positive or CAD negative, using a pre-specified cut-point. Finally, the continuous and binary outputs are compared to the ground truth labels to generate the reported performance of the algorithm, as described by ROC-AUC and sensitivity/specificity, respectively.

A proprietary signal capture device (CorVista Capture: Analytics for Life; Toronto, ON, Canada & Bethesda, MD, USA) acquired the orthogonal voltage gradient (OVG) from thorax electrodes, comprising three bipolar channels: front-rear, left-right, and top-bottom, as shown in Figure 1. Simultaneously, the device captured a photoplethysmogram (PPG) from a finger probe. The patient was supine and at rest. Signals were captured at 8 kHz for 215 s, packaged with a study-specific patient identifier, as well as patient height, weight, birth gender, and date of birth.

### 2.3. Machine-Learned Algorithm

The second-generation machine-learned algorithm for the detection of functionally significant CAD was developed using 290 features derived from the OVG and PPG signals [16,17,18]. Elastic Net and Random Forest were ensembled to generate candidate algorithms, which were assessed using cross-validation (five-fold) to optimize the parameters and select the final algorithm configuration. The algorithm cut-point, defining test positivity vs. negativity, was selected and locked during development. This final algorithm was embedded in a high-throughput processing system, which was then used to process the validation dataset. Further details on the model development process and its performance on the development data can be found in [14].

### 2.4. Validation Population Groups

**Figure 2:**
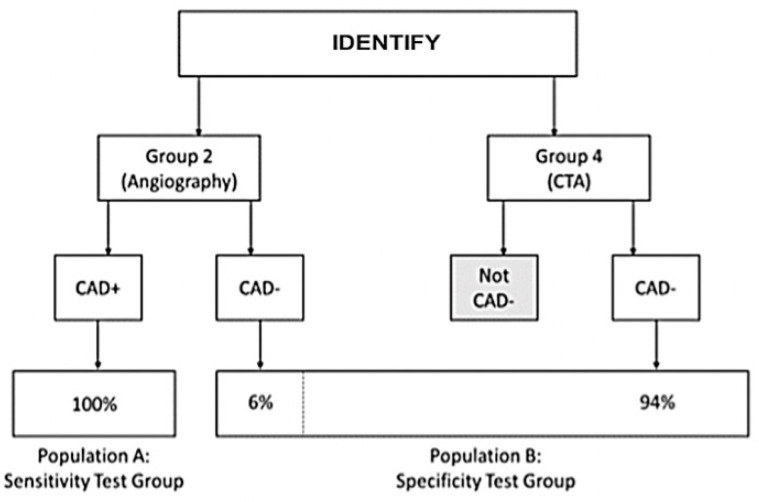
Construction of the validation population—Population A (sensitivity cohort) and Population B (specificity cohort), as derived from IDENTIFY.

The co-primary endpoints for algorithm validation assessed the validation population through the use of two distinct test groups: Population A for sensitivity and Population B for specificity, as shown in Figure 2. Signal acquisition for all subjects was performed within 7 days prior to the reference test (ICA/CCTA). 

Population A—Sensitivity Test Group: Cohort of subjects without any history of CAD and already scheduled for ICA to evaluate new onset symptoms consistent with CAD. This data set is constructed from subjects in IDENTIFY Group 2 for whom ICA results were available. Subjects were classified as CAD positive using the following criteria:

(I) a stenosis of ≥50% located in the LMCA,
(II) a stenosis of ≥70%, or FFR ≤0.80, or iFR ≤ 0.89, located in the LAD, LCX, RCA, ramus, or any of their distributions
(III) Functional assessment (FFR, iFR) superseded the lesion percentage when both were available.

Population B—Specificity Test Group: Cohort of subjects with new-onset symptoms suggestive of flow-limiting CAD with no known coronary artery disease. Note that current acute myocardial infarction (MI) and previous MI are excluded, and, therefore, this population does not contain MI with nonobstructive coronary arteries (MINOCA). These subjects were determined to not have CAD by meeting the criteria of one of the following groups:

(I) IDENTIFY Group 2 subjects who were identified as CAD negative by meeting none of the Population A criteria, as determined through assessment of the ICA report.
(II) IDENTIFY Group 4 subjects who underwent CCTA, with images overread by an independent core lab (Global Institute for Research (GIR), Midlothian, VA) and determined to be negative for significant CAD (CADRADS 0–2 and absent any recommendation for further assessment).

The defined groups were weighted using the below proportions for the primary objective analysis, as per the ratios in the PROMISE study [10]:

- IDENTIFY Group 2 at 6%, representing the portion of subjects that would be referred to ICA (10% of the original symptomatic population), which were subsequently determined to be CAD negative (60% of the ICA group).
- IDENTIFY Group 4 at 94%.

### 2.5. Statistics

The point performance and lower confidence bound for CCTA sensitivity have been reported as 0.93 and 0.89, and 0.53 and 0.37 for specificity [9]. Consequently, endpoints for this algorithm were set to demonstrate similar performance, with target point performance and lower confidence bound for sensitivity of 0.90 and 0.80 and those for specificity of 0.50 and 0.40. 

As this validation uses two independent co-endpoints (sensitivity and specificity), to power the validation with 81% confidence, each endpoint needs to be powered at 90%. Assuming a sensitivity point performance of 0.90 with a one-sided alpha of 0.025 and 90% power, at least 131 CAD-positive subjects were required. Assuming a specificity point performance of 0.50 with the same constraints, at least 260 CAD-negative subjects were required. To be included in the analysis, subjects must have met all inclusion criteria and no exclusion criteria, undergone their reference test (CCTA/ICA) within 7 days after signal collection, have had no major protocol deviations, and had a signal that passed outlier detection and quality checks. Failing any of the previous resulted in the exclusion of the subject. 

In addition to the primary co-endpoints of sensitivity and specificity, the receiver operator characteristic curve (ROC) and the corresponding area under the curve (ROC-AUC) were assessed. Subgroup performance was evaluated. The algorithm output was evaluated in tertiles within the test-negative and test-positive groups to determine the relevance of the value of the score beyond the binary result (further calculation details in Supplement Section S6). 

Clinical investigators did not have access to outputs from the machine-learned algorithm (was not acted upon clinically in this study), nor did technical staff invoking the algorithm have access to any ICA/CCTA results. The algorithm output and ICA/CCTA results were paired only by the third-party statistician (Technomics).

## 3. Results

A total of 1511 subjects were enrolled in Group 2, with CONSORT flow available in Supplement Section S4. Of these, 3% (*n* = 44) were excluded due to a major protocol deviation, including catheterization further than 7 days from signal collection and enrollment without meeting study entrance criteria. Additionally, there were <1% that either did not have ICA results available (*n* = 8) or the catheterization result was unable to be determined (*n* = 2). There were 47 subjects (3%) that did not have a signal received due to insufficient time to have the signal collection completed prior to the cardiac catheterization procedure or due to internet connection problems or improper device use (e.g., device not kept properly charged).

The remaining subjects (*n* = 1410) in Group 2 (Populations A and B) met all inclusion and exclusion criteria and did not have any major protocol deviations. Of these, 9.4% (*n* = 133) did not have passing signal quality, and 7.2% (*n* = 102) were deemed outlying by an outlier detection module. The remaining subjects (*n* = 1175) were used for validation. In particular, Group 2 Population A contained 488 (41.5%) subjects with significant CAD and were used for sensitivity testing. Of the 488, 300 (61%) of the subjects exhibited multi-vessel disease, and 188 (39%) had single-vessel disease. Group 2 Population B contained 687 (58.5%) subjects without significant CAD that were used for specificity testing. 

The Group 4 CONSORT flow is available in Supplement Section S5. Adjudication as either CAD negative or not CAD negative was conducted by an independent core lab. A total of 1246 subjects were enrolled in IDENTIFY Group 4. Of these, 4% (*n* = 51) had major protocol deviations. Additionally, 6% (*n* = 75) did not have imaging data available, and 28% (*n* = 356) of the subjects had CADRADS other than 0–2 or a referral for additional testing.

The remaining subjects (*n* = 764) in Group 4 Population B met all inclusion and exclusion criteria and did not have any major protocol deviations. Less than 1% (*n* = 8) did not have a signal. Of the remaining, 9.5% (*n* = 72) did not have a signal meeting signal quality requirements. Additionally, 5.6% (*n* = 43) had outlying signals. The remaining 641 subjects were used for specificity testing. Table 1 describes the demographic characteristics of Populations A and B. Distributions of age and BMI are provided in Supplement Section S8. A comparison of the key demographics between development and validation is provided in Supplement Section S7. Population A, across development and validation, showed significant differences in age, BMI, and hyperlipidemia. Population B, across development and validation, showed a significant difference in BMI. The significant changes across development and validation illustrate the enhanced difficulty of the date-based blinding strategy, as these differences would not be expected to appear by chance when using randomization. 

In summary, after removal for signal quality, outlier status, and major protocol deviations, Population A (sensitivity) was composed of 488 subjects, and Population B (specificity) was composed of 1328 subjects. The total of 1328 in Population B consisted of 687 determined to be CAD negative with ICA and 641 with CCTA. No treatment-emergent adverse events occurred during signal acquisition, nor CCTA/ICA. 

**Figure 3:**
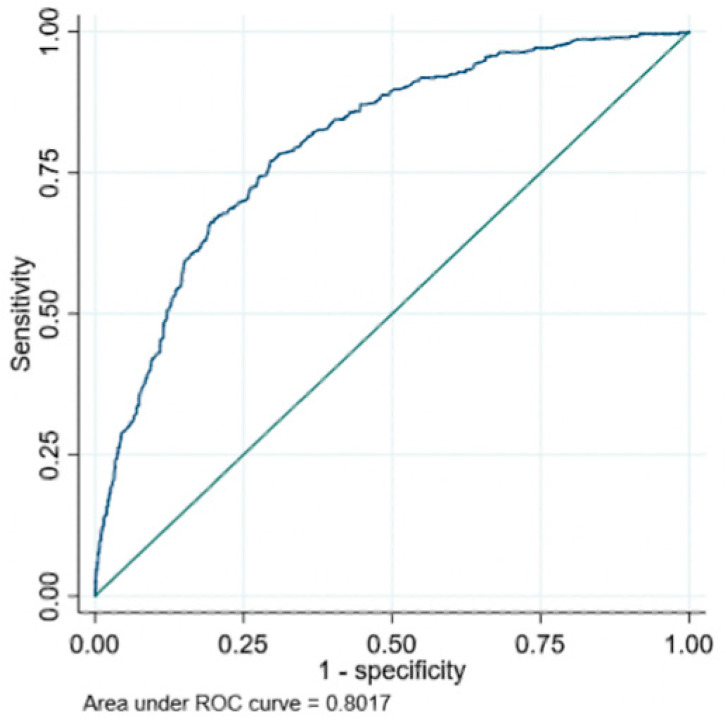
ROC curve showing model performance against the validation population.

Table 2 demonstrates that the null hypothesis can be rejected for both sensitivity and specificity and, therefore, the algorithm passes the pre-defined endpoints at the 95% confidence level. The performance in each component of the specificity population is also presented. The algorithm ROC-AUC was 0.80 (0.78–0.82), with the ROC curve presented in Figure 3.

Table 3 presents the subgroup performances. A significant difference (*p* < 0.01) in sensitivity was observed between males and females. Significant differences in specificity (*p* < 0.01) were observed between females and males by age (age ≥ 65 years vs. age < 65 years), hypertension status, and hyperlipidemia status.

A portion of the validation population has previously been used in the assessment of a previous-generation CAD algorithm [18]. The authors impose strict controls on the use and access to results from the validation dataset that could be used to tune future algorithms. To further demonstrate that no such knowledge has leaked across this boundary, the results of an assessment of the previously used vs. previously unused components of the validation population are also presented in Table 3.

When the algorithm test-negative scores were segmented into tertiles, a trend was observed of lower negative likelihood ratios in score ranges further away from zero as compared to closer to zero (Table 4). Inversely, the test-positive segmentation showed higher positive likelihood ratios further away from zero as compared to closer to zero (Table 4). 

## 4. Discussion

The results presented here were in a large, blinded population consisting of 1175 ICA subjects and 641 CCTA subjects. For comparison, one of the largest studies to date of CCTA and SPECT performance is the PROMISE study, which had an ICA population of 1015, representing approximately 10% of the incoming population (10,003) [10]. Recruiting patients already scheduled for either ICA or CCTA does not introduce bias into the sensitivity and specificity populations. Calculating sensitivity solely requires patients that can be identified as having significant CAD. The ICA population, by definition, provides all patients that can be identified as such when following the standard of care.

Calculating specificity solely requires patients who can be identified as not having significant CAD. A 6% weighted component of the ICA specificity population is added to match the ratios observed in the PROMISE study [10]. The specificity population, therefore, reflects the anticipated intended use population, comprising patients with symptoms of cardiovascular disease with no previous indication for significant CAD. The pre-specified endpoints were designed to demonstrate similar performance to CCTA as a rule-out test for CAD. With point performances for sensitivity of 0.85 and specificity of 0.58, and lower confidence bounds that passed the endpoints, this validation has demonstrated that the algorithm can be applied to the intended use population and deliver comparable performance to CCTA.

Minority groups and women are historically under-represented in cardiology [19]. The results show similar overall performance between males and females, with a different skew between sensitivity and specificity. Therefore, the overall algorithm performance is similar in both men and women, but the cut-point could be adjusted for females to align to the male performance profile. A cut-point in the female subgroup -0.045 lower than the pre-specified cut-point produces a sensitivity of 0.87 and specificity of 0.52, which is one point lower than males for sensitivity and two points higher for specificity, which are not significantly different. Given that females are underserved by current tests for CAD, implementing a solution that provides equal performance in both genders would be highly beneficial.

The algorithm specificity is lower in the population greater than 65 years old, whereas sensitivity is the same in both the older and younger groups. The algorithm specificity was also significantly different between those with/without hypertension as well as those with/without hyperlipidemia. In these cases, however, the differences were in terms of degree of exceeding the endpoints. Finally, the proportion of Black or African American subjects exceeded 15%, with no significant difference observed in algorithm performance between Black or African American subjects and White/Caucasian subjects.

Considering the modification of disease probability from pre-test to post-test when applying this algorithm, the likelihood ratios are LR+ = 2.02 and LR− = 0.26. As anticipated from the sensitivity and specificity, the likelihood ratios of the algorithm and CCTA are not dissimilar. Further, the continuous nature of the algorithm output enables further granularity to the test result beyond test-negative and test-positive. As described, the negative likelihood ratio is lower in more strongly negative ranges of the scores and vice versa in test-positive. Therefore, the assessment of the post-test probability of disease could be based on a more precise range of scores beyond simple test negativity/positivity. 

The specificity in the ICA component (Group 2) is lower than that in the CCTA component (Group 4). This is the logical consequence of removing truly negative patients, as identified by the standard of care prior to catheterization. An analysis of the performance of CCTA in filtered ICA populations shows that when only patients with lesions greater than 50% are referred to ICA, CCTA specificity is 0.25 (0.13–0.29) [20]. This is nearly identical to that observed in the IDENTIFY trial, with CCTA specificity being 0.26 (0.19–0.33) when the standard of care is used to refer patients to ICA. The algorithm performance in the ICA population, 0.21 (0.18–0.24), is directly comparable to these performances.

When analyzing the performance of machine-learned algorithms, it is critical to consider the biases, both intrinsic and extrinsic, that may potentially become integrated into the algorithm [19]. Typical issues include the heterogeneity in reference standard methodology between institutions and geographical differences in prevalence. The validation population encompasses areas with varying prevalence rates: higher (New York, Louisiana, Oklahoma, Mississippi, Texas), moderate (Florida, South Carolina), and comparatively lower (Georgia, North Carolina, Nebraska, Kansas) [21]. Therefore, the validation dataset is expected to represent the intended use population. Further, it was sourced from 20 distinct sites as another measure of bias reduction.

A potential machine-learning pitfall is overfitting [19]. The presented results represent performance on a blinded validation set consecutively enrolled after all development was completed. Further, there is also variation in staff and clinical sites between the development and validation datasets, which provides further confidence that the validation results are generalizable to the intended use population.

The results of an earlier-generation CAD algorithm were previously published [18]. Improvements that contributed to the higher performance of this generation included the use of different machine-learning methods and access to a larger volume of development data with equal weighting of males and females.

The first limitation of the algorithm presented herein is the imbalance in sensitivity and specificity between men and women despite equal overall performance. As discussed above, this potential limitation will be addressed in the commercial version of the algorithm through the implementation of an alternative cut-point for females, which corrects any sex-based difference in sensitivity/specificity performance. The second limitation is that the algorithm validation is limited to the population defined by the inclusion/exclusion criteria, and, therefore, no assessment or estimation of performance can be performed on populations not meeting these criteria. For instance, patients with prior (critical/non-critical) CAD were excluded, and, therefore, nothing is known about how the algorithm will perform on this cohort. However, this cohort is among those who may be targeted for future study. 

## 5. Conclusions

The previously developed algorithm was frozen and assessed in a static manner on a large blinded dataset and exhibited robust performance. The algorithm performance is comparable to CCTA, the standard of care rule-out test for CAD. However, unlike CCTA or SPECT, the results are available before the patient leaves the office (at the point of care), minimizing the fraction lost to follow-up. Rapid testing at the point of care is enabled by embedding the algorithm within the CorVista System, an FDA-cleared medical device comprising the CorVista Capture (signal acquisition), CorVista Analyzer (software to analyze the signal, including the CAD algorithm described herein), and CorVista Portal (web portal with which to access the results). The signals are transmitted from the CorVista Capture to the CorVista Analyzer automatically via WiFi or cellular connection, and results are returned on the CorVista Portal within 15 min. In rural areas, due to fear, scheduling limitations, and travel requirements, up to 50% of patients never complete the CCTA or SPECT test (personal communication, rural cardiologist), partially accounting for the significantly lower life expectancy in rural regions of the US. The performance was validated in a population modeling the intended use population, comprising symptomatic patients with no previous CAD diagnosis. Importantly, the overall performance in females is equal to that of males. This system addresses the need for a non-invasive, no-stress, no-radiation front-line test available at the point of care with significant advantages to the patient, physician, and healthcare system.

## Figures and Tables

**Table 1:** Demographic characteristics by population.

Characteristic	Population A (*n* = 488)	Population B (*n* = 1328)	Total (*n* = 1816)
Age at consent			
Mean ± SD	66.1 ± 9.2	58.3 ± 11.4	60.4 ± 11.4
<65	40.6% (198/488)	67.6% (898/1328)	60.4% (1096/1816)
≥65	59.4% (290/488)	32.4% (430/1328)	39.6% (720/1816)
Sex			
Female	30.9% (151/488)	56.3% (747/1328)	49.4% (898/1816)
Male	69.1% (337/488)	43.8% (581/1328)	50.6% (918/1816)
Ethnicity			
Not Hispanic or Latino	97.5% (473/485)	98.3% (1303/1326)	98.1% (1776/1811)
Hispanic or Latino	2.5% (12/485)	1.7% (23/1326)	1.9% (35/1811)
Race			
American Indian or Alaska Native	1.0% (5/482)	0.1% (1/1323)	0.3% (6/1805)
Asian	0.8% (4/482)	0.4% (5/1323)	0.5% (9/1805)
Black or African American	8.7% (42/482)	20.2% (267/1323)	17.1% (309/1805)
Native Hawaiian or Pacific Islander	0.4% (2/482)	0.3% (4/1323)	0.3% (6/1805)
White/Caucasian	87.3% (421/482)	77.2% (1022/1323)	79.9% (1443/1805)
Other	1.7% (8/482)	1.8% (24/1323)	1.8% (32/1805)
Prefer Not to Answer	1.2% (6/488)	0.4% (5/1328)	0.6% (11/1816)
BMI			
Mean ± SD	30.7 ± 6.1	32.9 ± 7.5	32.3 ± 7.2
<30	50.5% (246/487)	39.4% (523/1328)	42.4% (769/1815)
≥30	49.5% (241/487)	60.6% (805/1328)	57.6% (1046/1815)
Tobacco Use	56.6% (276/488)	46.2% (613/1328)	49.0% (889/1816)
Family History of Heart Attack	36.5% (178/488)	35.8% (476/1328)	36.0% (654/1816)
Hypertension	77.9% (380/488)	65.8% (874/1328)	69.1% (1254/1816)
Diabetes	38.9% (190/488)	24.0% (319/1328)	28.0% (509/1816)
Hypercholesterolemia/ Hyperlipidemia	83.6% (408/488)	63.6% (845/1328)	69.0% (1253/1816)

One subject did not have a height (and therefore, also BMI) recorded. BMI in (kg/m^2^). Tobacco use contains both past and present vs. never. Coloring identifies grouped rows.

**Table 2:** Primary endpoint results.

	CAD Positive Population A	CAD Negative Population B		
		Group 2	Group 4	Overall
Predicted CAD Positive	414	546	255	801
Predicted CAD Negative	74	141	386	527
Sensitivity	0.85 (0.82, 0.88) *p*-value = 0.002	n/a		
Specificity	n/a	0.21 (0.18, 0.24)	0.60 (0.56, 0.64)	0.58 (0.54, 0.62) *p*-value < 0.001

*p*-values are in comparison to the pre-determined endpoints of sensitivity > 0.80 and specificity > 0.40 (one-sided, α = 0.025, normal approximation). Overall specificity is the weighted combination of Group 2 (6%) and Group 4 (94%).

**Table 3:** Subgroup performance.

Subgroup	Sensitivity (95% CI) (n/N)	*p*-Value	G2 Specificity (n/N)	G4 Specificity (n/N)	(6% G2, 94% G4) Overall Specificity (95% CI)	*p*-Value
Female	0.77 (0.70–0.84) (117/151)	0.002	0.24 (82/336)	0.65 (266/411)	0.63 (0.59–0.67)	0.001
Male	0.88 (0.85–0.91) (297/337)		0.17 (59/351)	0.52 (120/230)	0.50 (0.44–0.56)	
BMI ≥ 30	0.85 (0.80–0.90) (204/241)	1.000	0.21 (90/429)	0.61 (229/376)	0.59 (0.54–0.64)	0.592
BMI < 30	0.85 (0.81–0.89) (209/246)		0.20 (51/258)	0.59 (157/265)	0.57 (0.51–0.63)	
Age ≥ 65	0.87 (0.83–0.91) (251/290)	0.131	0.14 (40/291)	0.48 (67/139)	0.46 (0.38–0.54)	<0.001
Age < 65	0.82 (0.77–0.87) (163/198)		0.26 (101/396)	0.64 (319/502)	0.62 (0.58–0.66)	
Diabetic	0.87 (0.82–0.92) (166/190)	0.231	0.18 (36/196)	0.54 (66/123)	0.52 (0.44–0.60)	0.062
Non-Diabetic	0.83 (0.79–0.87) (246/296)		0.21 (104/489)	0.62 (320/518)	0.60 (0.56–0.64)	
Hypertensive	0.84 (0.80–0.88) (321/380)	0.609	0.18 (89/494)	0.54 (207/380)	0.52 (0.47–0.57)	<0.001
Non-Hypertensive	0.86 (0.79–0.93) (93/108)		0.27 (51/192)	0.69 (179/261)	0.66 (0.61–0.71)	
Hyperlipidemic	0.85 (0.82–0.88) (347/408)	0.820	0.18 (83/470)	0.56 (210/375)	0.54 (0.49–0.59)	0.009
Non-Hyperlipidemic	0.84 (0.76–0.92) (67/80)		0.27 (58/215)	0.66 (176/266)	0.64 (0.59–0.69)	
White/Caucasian	0.85 (0.82–0.88) (357/421)	0.732	0.20 (115/571)	0.62 (281/451)	0.59 (0.55–0.63)	0.191
Black or African American	0.83 (0.72–0.94) (35/42)		0.21 (19/92)	0.55 (96/175)	0.53 (0.46–0.60)	
Tobacco Use (Past/Present)	0.83 (0.79–0.87) (228/276)	0.1266	0.16 (55/343)	0.56 (151/270)	0.54 (0.48–0.60)	0.0563
Tobacco Use (Never)	0.88 (0.84–0.92) (186/212)		25 (86/344)	0.63 (235/371)	0.61 (0.56–0.66)	
Used in Previous Validation	0.85 (0.81–0.89) (209/246)	1.000	0.24 (88/374)	0.54 (114/213)	0.52 (0.46–0.58)	0.017
Not Used in Previous Validation	0.85 (0.80–0.90) (205/242)		0.17 (53/313)	0.64 (272/428)	0.61 (0.57–0.65)	

BMI is in kg/m. *p*-value calculated using the normal approximation test (two-sided). Confidence interval from normal approximation. Coloring identifies grouped rows.

**Table 4:** Likelihood ratios by tertiles.

Test-Negative		
Tertile	Score Range	Negative Likelihood Ratio
1st	(−0.446, −0.142)	0.118
2nd	(−0.141, −0.068)	0.265
3rd	(−0.065, −0.001)	0.366
**Test-Positive**		
**Tertile**	**Score Range**	**Positive Likelihood Ratio**
1st	(0.000, 0.094)	1.507
2nd	(0.095, 0.191)	2.289
3rd	(0.192, 0.619)	3.639

## Data Availability

Anonymized subsets of the dataset may be provided to academic researchers on a case-by-case basis.

## Supplementary Materials

The following supporting information can be downloaded at: https://www.mdpi.com/article/10.3390/diagnostics14100987/s1, Section S1: STARD Checklist; Section S2: Inclusion/Exclusion Criteria; Section S3: Clinical study sites and locations; Section S4: CONSORT Flow for IDENTIFY Group 2; Section S5: CONSORT Flow for IDENTIFY Group 4; Section S6: Relevance of CAD Score Beyond Binary Results. Section S7: Comparison Between Development and Validation Key Demographics; Section S8: Distribution of Age and BMI in the Validation Population.

## References

[1] NC Rural Health Research Program (UNC) (2017). Rural Health Snapshot (2017). https://www.shepscenter.unc.edu//wp-content/uploads/dlm_uploads/2017/05/Snapshot2017.pdf.

[2] U.S. Government Accountability Office (2023). Why Healthcare Is Harder to Access in Rural America. https://www.gao.gov/blog/why-health-care-harder-access-rural-america.

[3] Robin Warshaw (AAMC) (2017). Health Disparities Affect Millions in Rural U.S. Communities..

[4] Motairek I., Chen Z., Makhlouf M.H., Deo S., Salerno P.R., Mentias A., Nasir K., Rajagopalan S., Al-Kindi S.G. (2023). Mapping geographic proximity to cardiologists across the United States. Circ. Cardiovasc. Qual. Outcomes.

[5] Bossard M., Latifi Y., Fabbri M., Kurmann R., Brinkert M., Wolfrum M., Berte B., Cuculi F., Toggweiler S., Kobza R. (2020). Increasing mortality from premature coronary artery disease in women in the rural United States. J. Am. Heart Assoc..

[6] Fihn S.D., Gardin J.M., Abrams J., Berra K., Blankenship J.C., Dallas P., Douglas P.S., Foody J.M., Gerber T.C., Hinderliter A.L. (2012). 2012 ACCF/AHA/ACP/AATS/PCNA/SCAI/STS guideline for the diagnosis and management of patients with stable ischemic heart disease: A report of the American College of Cardiology Foundation/American Heart Association task force on practice guidelines, and the American College of Physicians, American Association for Thoracic Surgery, Preventive Cardiovascular Nurses Association, Society for Cardiovascular Angiography and Interventions, and Society of Thoracic Surgeons. Circulation.

[7] Gianrossi R., Detrano R., Mulvihill D., Lehmann K., Dubach P., Colombo A., McArthur D., Froelicher V. (1989). Exercise-induced ST depression in the diagnosis of coronary artery disease. A meta-analysis. Circulation.

[8] Gulati M., Levy P.D., Mukherjee D., Amsterdam E., Bhatt D.L., Birtcher K.K., Blankstein R., Boyd J., Bullock-Palmer R.P., Conejo T. (2021). 2021 AHA/ACC/ASE/CHEST/SAEM/SCCT/SCMR guideline for the evaluation and diagnosis of chest pain: A report of the American College of Cardiology/American Heart Association Joint Committee on Clinical Practice Guidelines. J. Am. Coll. Cardiol..

[9] Knuuti J., Ballo H., Juarez-Orozco L.E., Saraste A., Kolh P., Rutjes A.W.S., Jüni P., Windecker S., Bax J.J., Wijns W. (2018). The performance of non-invasive tests to rule-in and rule-out significant coronary artery stenosis in patients with stable angina: A meta-analysis focused on post-test disease probability. Eur. Heart J..

[10] Douglas P.S., Hoffmann U., Patel M.R., Mark D.B., Al-Khalidi H.R., Cavanaugh B., Cole J., Dolor R.J., Fordyce C.B., Huang M. (2015). Outcomes of anatomical versus functional testing for coronary artery disease. N. Engl. J. Med..

[11] Patel M.R., Peterson E.D., Dai D., Brennan J.M., Redberg R.F., Anderson H.V., Brindis R.G., Douglas P.S. (2010). Low diagnostic yield of elective coronary angiography. N. Engl. J. Med..

[12] Delshad J., Makaryus A. Less than half of safety-net hospitals across the US provide CCTA for the evaluation of cardiac patients. Proceedings of the SCCT.

[13] Pelletier-Galarneau M., Vandenbroucke E., Lu M., Li O. (2023). Characteristics and key differences between patient populations receiving imaging modalities for coronary artery disease diagnosis in the US. BMC Cardiovasc. Disord..

[14] Burton T., Fathieh F., Nemati N., Gillins H.R., Shadforth I.P., Ramchandani S., Bridges C.R. (2024). Development of a Non-Invasive Machine-Learned Point-Of- Care Rule-Out Test for Coronary Artery Disease. Diagnostics.

[15] Cohen J.F., Korevaar D.A., Altman D.G., Bruns D.E., Gatsonis C.A., Hooft L., Irwig L., Levine D., Reitsma J.B., De Vet H.C. (2016). STARD 2015 guidelines for reporting diagnostic accuracy studies: Explanation and elaboration. BMJ Open.

[16] Fathieh F., Paak M., Khosousi A., Burton T., Sanders W.E., Doomra A., Lange E., Khedraki R., Bhavnani S., Ramchandani S. (2021). Predicting cardiac disease from interactions of simultaneously-acquired hemodynamic and cardiac signals. Comput. Methods Programs Biomed..

[17] Bhavnani S.P., Khedraki R., Cohoon T.J., Meine F.J., Stuckey T.D., McMinn T., Depta J.P., Bennett B., McGarry T., Carroll W. (2022). Multicenter validation of a machine learning phase space electro-mechanical pulse wave analysis to predict elevated left ventricular end diastolic pressure at the point-of-care. PLoS ONE.

[18] Stuckey T., Meine F., McMinn T., Depta J.P., Bennett B., McGarry T., Carroll W., Suh D., Steuter J.A., Roberts M. (2022). Development and validation of a machine learned algorithm to IDENTIFY functionally significant coronary artery disease. Front. Cardiovasc. Med..

[19] Tat E., Bhatt D.L., Rabbat M.G. (2020). Addressing bias: Artificial intelligence in cardiovascular medicine. Lancet Digit. Health.

[20] Koo B.K., Erglis A., Doh J.H., Daniels D.V., Jegere S., Kim H.S., Dunning A., DeFrance T., Lansky A., Leipsic J. (2011). Diagnosis of ischemia-causing coronary stenoses by noninvasive fractional flow reserve computed from coronary computed tomographic angiograms: Results from the prospective multicenter DISCOVER-FLOW (Diagnosis of Ischemia-Causing Stenoses Obtained Via Noni. J. Am. Coll. Cardiol..

[21] Roth G.A., Johnson C.O., Abate K.H., Abd-Allah F., Ahmed M., Alam K., Alam T., Alvis-Guzman N., Ansari H., Ärnlöv J. (2018). The burden of cardiovascular diseases among US states, 1990–2016. JAMA Cardiol.

