# Supplementary material for: Clinical Validation of a Machine-Learned, Point-of-Care System to IDENTIFY Functionally Significant Coronary Artery Disease
Source: Diagnostics (Basel). 2024 May 8;14(10):987. doi: 10.3390/diagnostics14100987 (PMC11120588; doi:10.3390/diagnostics14100987)
Supplement: Supplementary file 1 [file diagnostics-14-00987-s001.zip › diagnostics-2971459-supplementary.pdf]

# Supplementary Section S1 – STARD Checklist

Table S1 - STARD Checklist.

| Section & Topic | No | Item | **Reported on page #** |
|---|---|---|---|
| TITLE OR ABSTRACT | | | |
| | 1 | Identification as a study of diagnostic accuracy using at least one measure of accuracy (such as sensitivity, specificity, predictive values, or AUC) | 1 |
| ABSTRACT | | | |
| | 2 | Structured summary of study design, methods, results, and conclusions (for specific guidance, see STARD for Abstracts) | 1 |
| INTRODUCTION | | | |
| | 3 | Scientific and clinical background, including the intended use and clinical role of the index test | 2-3 |
| | 4 | Study objectives and hypotheses | 3 |
| METHODS | | | |
| Study design | 5 | Whether data collection was planned before the index test and reference standard were performed (prospective study) or after (retrospective study) | 4 |
| Participants | 6 | Eligibility criteria | 3, Supplement Section S2 |
| | 7 | On what basis potentially eligible participants were identified (such as symptoms, results from previous tests, inclusion in registry) | 3-5 |
| | 8 | Where and when potentially eligible participants were identified (setting, location and dates) | Supplement Section S3 |
| | 9 | Whether participants formed a consecutive, random or convenience series | 3 |
| Test methods | 10a | Index test, in sufficient detail to allow replication | 4 |
| | 10b | Reference standard, in sufficient detail to allow replication | 4-5 |
| | 11 | Rationale for choosing the reference standard (if alternatives exist) | 2-3 |
| | 12a | Definition of and rationale for test positivity cut-offs or result categories of the index test, distinguishing pre-specified from exploratory | 4 |
| | 12b | Definition of and rationale for test positivity cut-offs or result categories of the reference standard, distinguishing pre-specified from exploratory | 5 |
| | 13a | Whether clinical information and reference standard results were available to the performers/readers of the index test | 6 |
| | 13b | Whether clinical information and index test results were available to the assessors of the reference standard | 6 |
| Analysis | 14 | Methods for estimating or comparing measures of diagnostic accuracy | 5-6 |
| | 15 | How indeterminate index test or reference standard results were handled | 5-6 |
| | 16 | How missing data on the index test and reference standard were handled | 5-6 |
| | 17 | Any analyses of variability in diagnostic accuracy, distinguishing pre-specified from exploratory | n/a |
| | 18 | Intended sample size and how it was determined | 6 |
| RESULTS | | | |
| Participants | 19 | Flow of participants, using a diagram | Supplement Section S5 & S6 |
| | 20 | Baseline demographic and clinical characteristics of participants | Table 1 |
| | 21a | Distribution of severity of disease in those with the target condition | 6 |
| | 21b | Distribution of alternative diagnoses in those without the target condition | Table 1 |
| | 22 | Time interval and any clinical interventions between index test and reference standard | 5-6 |
| Test results | 23 | Cross tabulation of the index test results (or their distribution) by the results of the reference standard | Table 2 |
| | 24 | Estimates of diagnostic accuracy and their precision (e.g. 95% confidence intervals) | 8, Table 2 |
| | 25 | Any adverse events from performing the index test or the reference standard | 7 |
| DISCUSSION | | | |
| | 26 | Study limitations, including sources of potential bias, statistical uncertainty, and generalizability | 12 |
| | 27 | Implications for practice, including the intended use and clinical role of the index test | 12 |
| OTHER INFORMATION | | | |
| | 28 | Registration number and name of registry | 3 |
| | 29 | Where the full study protocol can be accessed | 3 |
| | 30 | Sources of funding and other support; role of funders | 12 |

# Supplementary Section S2 – Inclusion/Exclusion Criteria

Table S2 - Inclusion/Exclusion Criteria.

| Inclusion | Exclusion |
|---|---|
| ≥ 18 years of age | Prior documented history of myocardial infarction (MI) |
| Cardiovascular symptoms | Suspected acute myocardial infarction (AMI) at current presentation |
| Scheduled to undergo cardiac catheterization with coronary angiography (Group 2) or Computed Tomography Angiography (Group 4) | Prior coronary artery bypass grafting (CABG) |
| Ability to understand the requirements of the study and to provide written informed consent | Prior heart valve replacement |
| | Previous sustained or paroxysmal atrial or ventricular arrythmia |
| | Infiltrative myocardial disease (amyloid, sarcoid, right ventricular dysplasia) |
| | Presence of cardiac implantable electronic device (CIED), including implantable cardioverter defibrillator (ICD), pacemaker (PM), implantable loop recorders and other monitors |
| | Implantable Neuro-stimulators |
| | Congenital Heart Disease |
| | Pregnant or breast feeding |
| | Currently taking any Type IA, IC or III antiarrhythmics |
| | Any history of amiodarone use |
| | Clinically significant chest deformity (e.g., pectus excavatum or pectus carinatum) |
| | Breast implants |
| | Neuromuscular disease if the condition results in tremor or muscle fasciculations |

# Supplemental Section S3 – Clinical study sites and locations.

Table S3 - Clinical study sites and locations.

| Site Name | Location | Site Used in Development | Site Used in Validation |
|---|---|---|---|
| Atlanta Heart Specialists | Tucker, GA | Yes | Yes |
| Cone Health Heart and Vascular Center | Greensboro, NC | Yes | Yes |
| Novant Health New Hanover Regional Medical Center | Wilmington, NC | Yes | Yes |
| Rochester General Hospital | Rochester, NY | Yes | Yes |
| Bryan Heart | Lincoln, NE | Yes | Yes |
| Piedmont Healthcare | Atlanta, GA | Yes | Yes |
| Austin Heart | Austin, TX | Yes | Yes |
| Cardiovascular Associates of the Southeast | Birmingham, AL | Yes | No |
| Sentara Hospital and Medical Group | Norfolk, VA | Yes | Yes |
| North Georgia Medical Center | Gainesville, GA | Yes | No |
| Jacobs Institute | Buffalo, NY | Yes | No |
| Lexington Medical Center Heart & Vascular | West Columbia, SC | Yes | Yes |
| Ochsner Medical Foundation | Jefferson, LA | Yes | No |
| Advent Health Tampa | Tampa, FL | No | Yes |
| Cardiovascular Institute of the South (Houma) | Houma, LA | Yes | Yes |
| Cardiovascular Institute of the South (Lafayette) | Lafayette, LA | Yes | Yes |
| Jackson Heart Clinic | Jackson, MS | Yes | Yes |
| Oklahoma Heart Hospital | Oklahoma City, OK | Yes | Yes |
| WellStar Research Institute | Marietta, GA | Yes | Yes |
| Cardiology Associates of North Mississippi | Tupelo, MS | Yes | Yes |
| Medical University of South Carolina | Charleston, SC | No | Yes |
| Minneapolis Heart | Minneapolis, MN | No | Yes |
| Alamance Regional Medical Center | Burlington, NC | No | Yes |
| Lundquist Institute | Torrance, CA | No | Yes |

## Supplemental Section S4 – CONSORT Flow for IDENTIFY Group 2

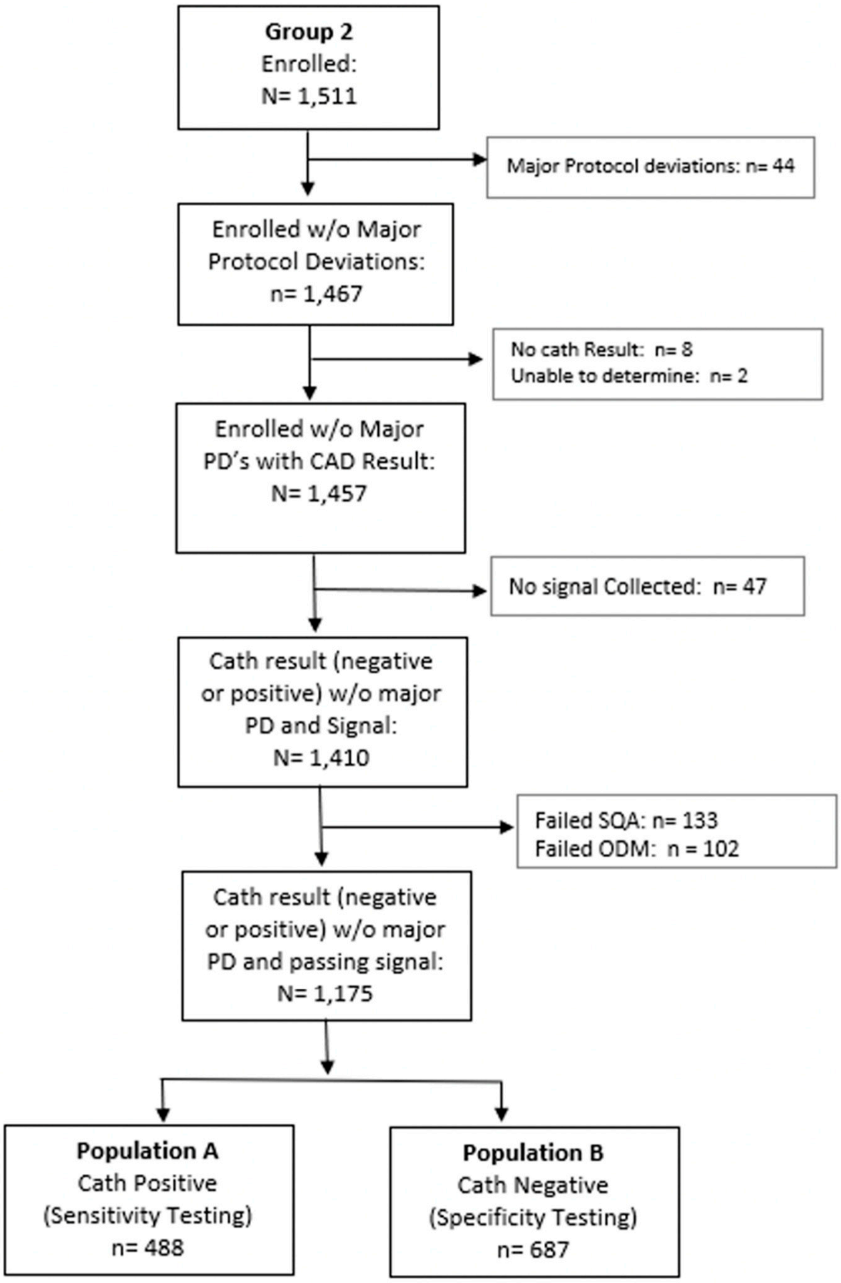


**Figure S1** - CONSORT Flow for IDENTIFY Group 2.

# Supplemental Section S5 – CONSORT Flow for IDENTIFY Group 4

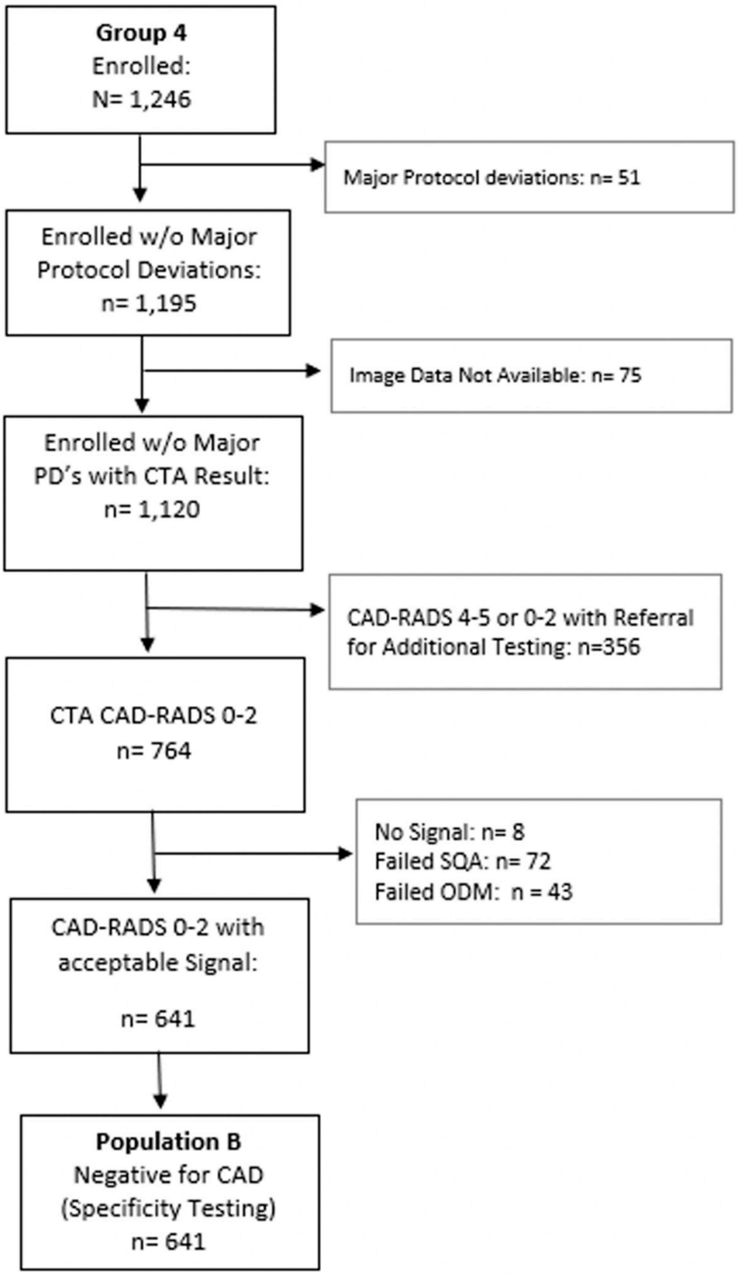


**Figure S2** - CONSORT Flow for IDENTIFY Group 4

## Supplemental Section S6 – Relevance of CAD Score Beyond Binary Results

The test-negative and test-positive ranges of the CAD Score were divided into tertiles (i.e., three ranges, each with an equal number of subjects). For this analysis, the Group 2 subjects in Population B were excluded, due to the minimal effect on specificity (Group 4 specificity of 60% is not substantially affected when including the Group 2 subjects, reducing only to 58%).

The negative and positive likelihood ratios were calculated for each tertile assuming that the score range represents the entire test-negative or test-positive range, respectively.

The results with the unmodified thresholds are show in Table 4. The following tables reflect the adjustment of the female threshold described in the discussion.

Table S4 – Score ranges and likelihood ratios for test-negative and test-positives for each tertile.

| Test-Negative | | |
|---|---|---|
| Tertile | Score Range | Negative Likelihood Ratio |
| Tertile 1 | (-0.401, -0.122) | 0.129 |
| Tertile 2 | (-0.121, -0.058) | 0.220 |
| Tertile 3 | (-0.057, -0.001) | 0.340 |
| **Test-Positive** | | |
| Tertile | Score Range | Positive Likelihood Ratio |
| Tertile 1 | (0.000, 0.100) | 1.446 |
| Tertile 2 | (0.101, 0.199) | 1.913 |
| Tertile 3 | (0.200, 0.644) | 2.888 |

# Supplemental Section S7 – Comparison Between Development and Validation Key Demographics

Table S5 – Comparison of Population A

| Characteristic | Development (n=641) | Validation (n=488) | p-value |
|---|---|---|---|
| Age at consent | | | |
| Mean ± SD | 64.9 ± 9.6 | 66.1 ± 9.2 | 0.0344 |
| <65 | 43.7% (280/641) | 40.6% (198/488) | 0.3240 |
| ≥65 | 56.3% (361/641) | 59.4% (290/488) | |
| Sex | | | |
| Female | 26.7% (171/641) | 30.9% (151/488) | 0.1321 |
| Male | 73.3% (470/641) | 69.1% (337/488) | |
| BMI | | | |
| Mean ± SD | 30.9 ± 6.2 | 30.7 ± 6.1 | 0.5888 |
| <30 | 45.7% (293/641) | 50.5% (246/487) | <0.05 |
| ≥30 | 54.3% (348/641) | 49.5% (241/487) | |
| Hypertension | 78.6% (504/641) | 77.9% (380/488) | 0.8155 |
| Diabetes | 34.5% (221/641) | 38.9% (190/488) | 0.1390 |
| Hypercholesterolemia/ Hyperlipidemia | 76.6% (491/641) | 83.6% (408/488) | 0.0048 |

Table S6 – Comparison of Population B

| Characteristic | Development (n=513) | Validation (n=641)* | p-value |
|---|---|---|---|
| Age at consent | | | |
| Mean ± SD | 55.0 ± 12.0 | 54.7 ± 11.3 | 0.6630 |
| <65 | 77.0% (395/513) | 78.3% (502/641) | 0.6432 |
| >=65 | 23.0% (118/513) | 21.7% (139/641) | |
| Sex | | | |
| Female | 64.5% (331/513) | 64.1% (411/641) | 0.9359 |
| Male | 35.5% (182/513) | 35.9% (230/641) | |
| BMI | | | |
| Mean ± SD | 31.3 ± 6.6 | 32.4 ± 7.1 | 0.0071 |
| <30 | 47.8% (245/513) | 41.3% (265/641) | 0.0339 |
| >=30 | 52.2% (268/513) | 58.7% (376/641) | |
| Hypertension | 60.0% (308/513) | 59.3% (380/641) | 0.8415 |
| Diabetes | 15.8% (81/513) | 19.2% (123/641) | 0.1537 |
| Hypercholesterolemia/ Hyperlipidemia | 52.6% (270/513) | 58.5% (375/641) | 0.0528 |

* CCTA (Group 4) only, since subjects without CAD from ICA were not used in training.

# Supplementary Section S8 – Distribution of Age and BMI in the Validation Population

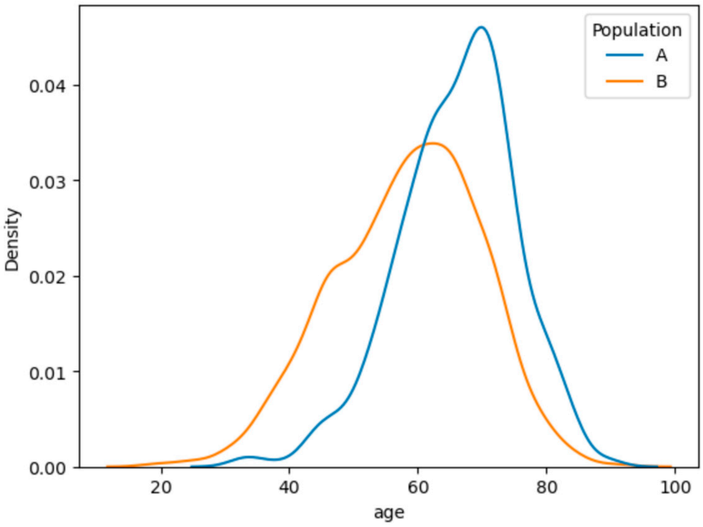


**Figure S3** – Distribution of Age

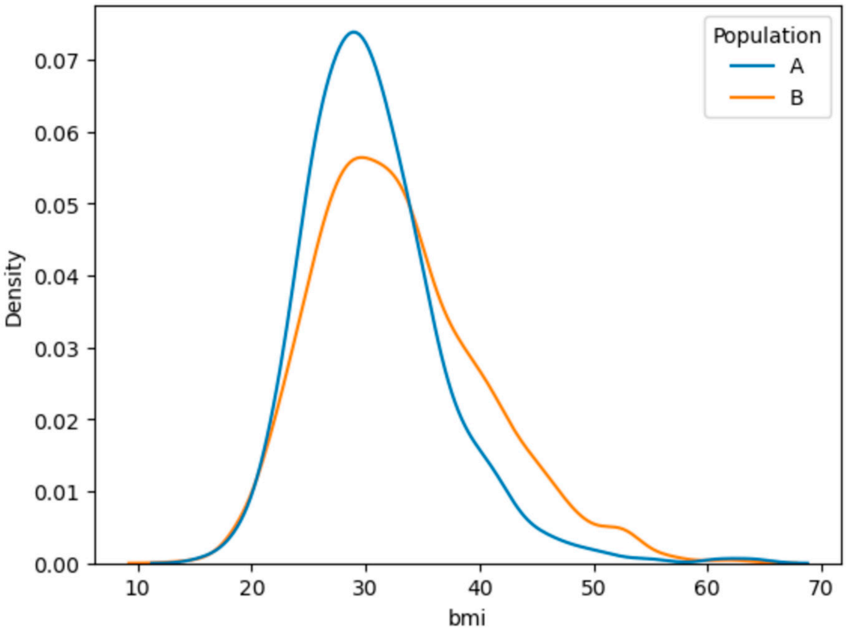


**Figure S4** – Distribution of BMI
